# Enhanced weathering as a trigger for the rise of atmospheric O_2_ level from the late Ediacaran to the early Cambrian

**DOI:** 10.1038/s41598-019-47142-3

**Published:** 2019-07-23

**Authors:** Wei-Ping Li, Yan-Yan Zhao, Ming-Yu Zhao, Xiang-Ping Zha, Yong-Fei Zheng

**Affiliations:** 1 CAS Key Laboratory of Crust-Mantle Materials and Environments, School of Earth and Space Sciences, University of Science and Technology of China, Hefei, 230026 China; 2 Key Laboratory of Submarine Geosciences and Prospecting Techniques, Ministry of Education, Institute for Advanced Ocean Study, College of Marine Geosciences, Ocean University of China, Qingdao, 266100 China; 3 Laboratory for Marine Mineral Resources, Qingdao National Laboratory for Marine Science and Technology, Qingdao, 266237 China; 4 Department of Geology and Geophysics, Yale University, New Haven, Connecticut 06511 USA

**Keywords:** Geochemistry, Sedimentology

## Abstract

A shift toward a higher oxygen level in both ocean and atmosphere systems during the late Ediacaran to the early Cambrian has been suggested from multiple indirect proxies. However, the mechanism and magnitude of this oxidation remain unclear. To solve this issue, we measured carbon isotopes in both carbonate and organic matter as well as their trace element compositions for an Ediacaran-Cambrian sequence in the Lower Yangtze basin, South China. The δ^13^C_org_ and δ^13^C_carb_ excursions of this sequence are coupled and can be compared with contemporaneous global carbon isotope curves. A 2‰ rise in Δ^13^C_carb-org_ occurred from the late Ediacaran to the early Cambrian, suggesting a substantial increase in atmospheric oxygen level from 16% to 30% of the present atmospheric level (PAL). Furthermore, the distribution pattern of rare earth elements and the concentrations of water-insoluble elements in the carbonates indicate a sudden enhancement in chemical weathering of the continental crust during the early Cambrian, which may be a trigger for the rise of atmospheric O_2_ level. Both the supply of a large amount of nutrients due to the enhanced continental weathering and the contemporary increase of atmospheric oxygen concentrations may have promoted the appearance of large metazoans in the early Cambrian.

## Introduction

Fluctuations in atmospheric oxygen (O_2_) and seawater redox play a fundamental role in driving biological evolution throughout the geological time^1,2^. Atmospheric and seawater dissolved oxygen levels increase during the growth of organic carbon reservoirs because the CO_2_ flux into the atmosphere–ocean system is balanced by carbon burial with the release of oxygen^3,4^. The Cambrian bioradiation is thought to have occurred when the dissolved oxygen concentrations in the seawater exceed the relatively high oxygen requirements of animals^5^. Although multiple lines of evidence demonstrate a short-lived return to widespread seawater anoxia at the Ediacaran-Cambrian (E-C) boundary^6–12^, evidence from sedimentary Mo concentrations^5,13,14^ and isotopes^15^, U isotopes^16,17^ and rare earth element concentrations^18^ demonstrates that the global seawater became progressively oxygenated in the early Cambrian^13,15,17,19–21^. The mechanism and magnitude of raising atmospheric oxygen concentrations, however, remain to be fundamental yet unresolved questions.

A rise in atmospheric oxygen concentrations can lead to chemical changes in the Cambrian seawater and trigger the metazoan evolution^15,22–25^. However, the high level of atmospheric oxygen alone cannot explain the rapid rise in the biological complexity of Cambrian age^26^. The supply of essential nutrients, such as P, Ca, Fe, Na^+^, K^+^ and Mo, were also considered to have exerted a significant control on the evolution of myriad life forms^21,27,28^. It has been suggested that a suddenly enhanced weathering of the continental crust during the E-C transition acted as a trigger for the rise in atmospheric oxygen concentrations^29–31^. The enhanced weathering and the rise of O_2_ may pave the way for the development of large and complex Cambrian animals. Here, we present the geochemical proxies of carbonates, such as the carbon isotope compositions, rare earth elements (REE + Y) distribution patterns and water-insoluble element concentrations, to track the mechanism and magnitude of atmospheric oxygen fluctuation during the early Cambrian.

**Figure 1:**
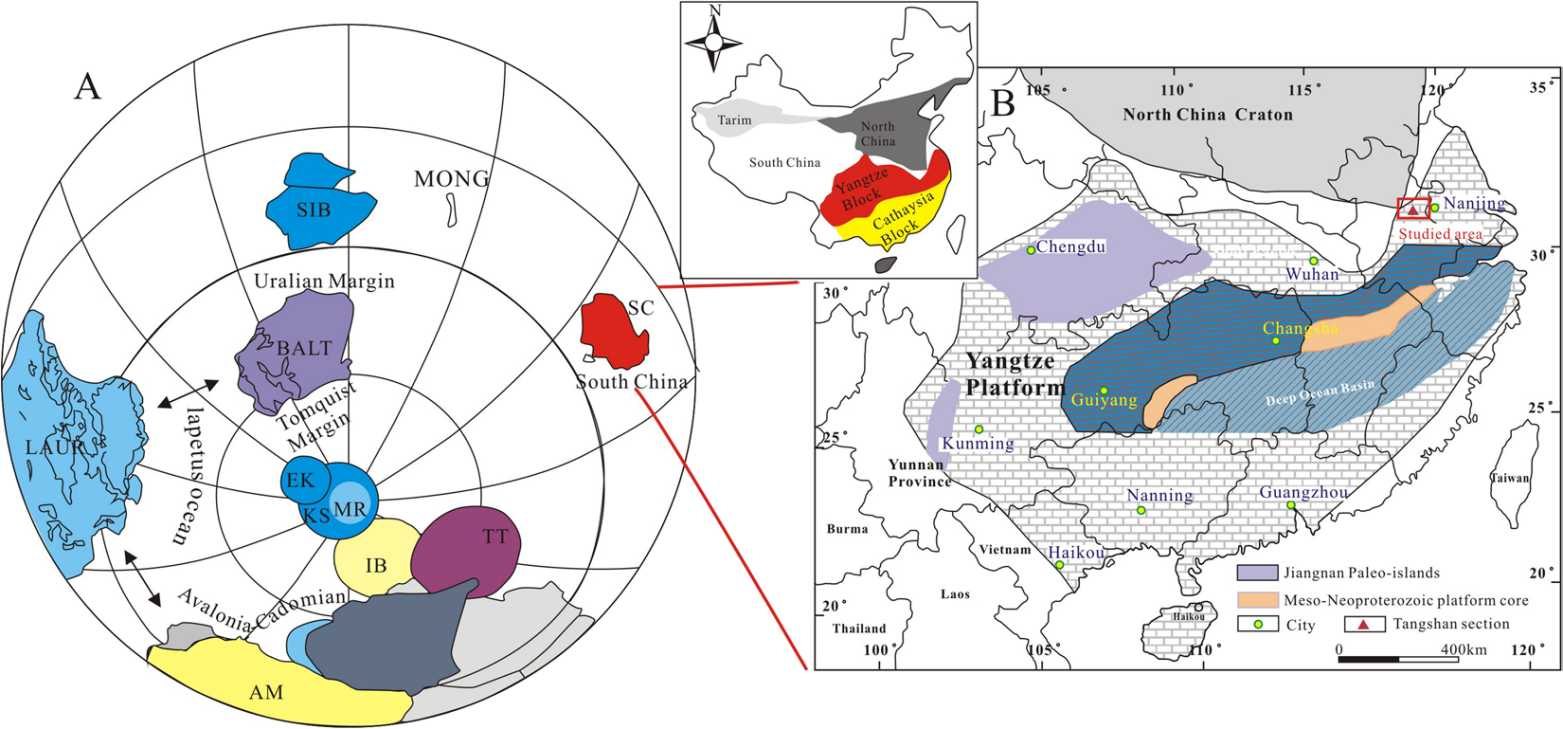
(**A**) Tentative paleogeographic reconstruction for ca. 542 Ma (modified after Maloof *et al*.^33^). Cratons are labeled: LAUR—Laurentia, AM—Amazonia, WA—West Africa, BALT—Baltica, SIB—Siberia, MONG—Mongolia, and SC—South China. (**B**) Depositional environments during the Ediacaran-Cambrian transition on the Yangtze platform and geological settings of the Tangshan section at Chaohu in eastern Anhui, South China (modified after Li *et al*.^40^). The insert map is the location of Yangtze platform and Cathaysia foldbelt. Red box and triangle denote the studied area and sample section, respectively.

The target carbonates were sampled from an Ediacaran-Cambrian sequence at the Tangshan section in the Lower Yangtze basin, South China (Fig. 1). This region belongs to part of the Jiangnan orogen that is located between the Yangtze platform in the northwest and the Cathaysia foldbelt in the southeast^32^. During the E-C transition, the Yangtze platform was located at low to middle latitudes in the Northern Hemisphere (Fig. 1A)^33^. It was evolved from a passive continental margin basin in the early Neoproterozoic to a series of rift basins in the middle Neoproterozoic, with a continental shelf to a marine basin from the northwest to the southeast^32^. The carbonate platform was surrounded by narrow marginal transitional zones to the north (~800 km long) and to the southwest (>1600 km long)^9,34,35^, along which shallow-water carbonates sharply change into basinward black chert/shale successions^35–37^. These sedimentary sequences on the continental shelf are characterized by repeated transgression–regression events^36^. The post-Fortunian lower Cambrian on the Yangtze platform, from the shelf (i.e. Xiaotan, Shantan, Jinsha and Weng’an) to the slope (i.e. Songtao and Longbizui), started with black shale with similar lithologic markers or/and the first appearance of the trilobite *T*. *niutitangensis*^9,38^.

**Figure 2:**
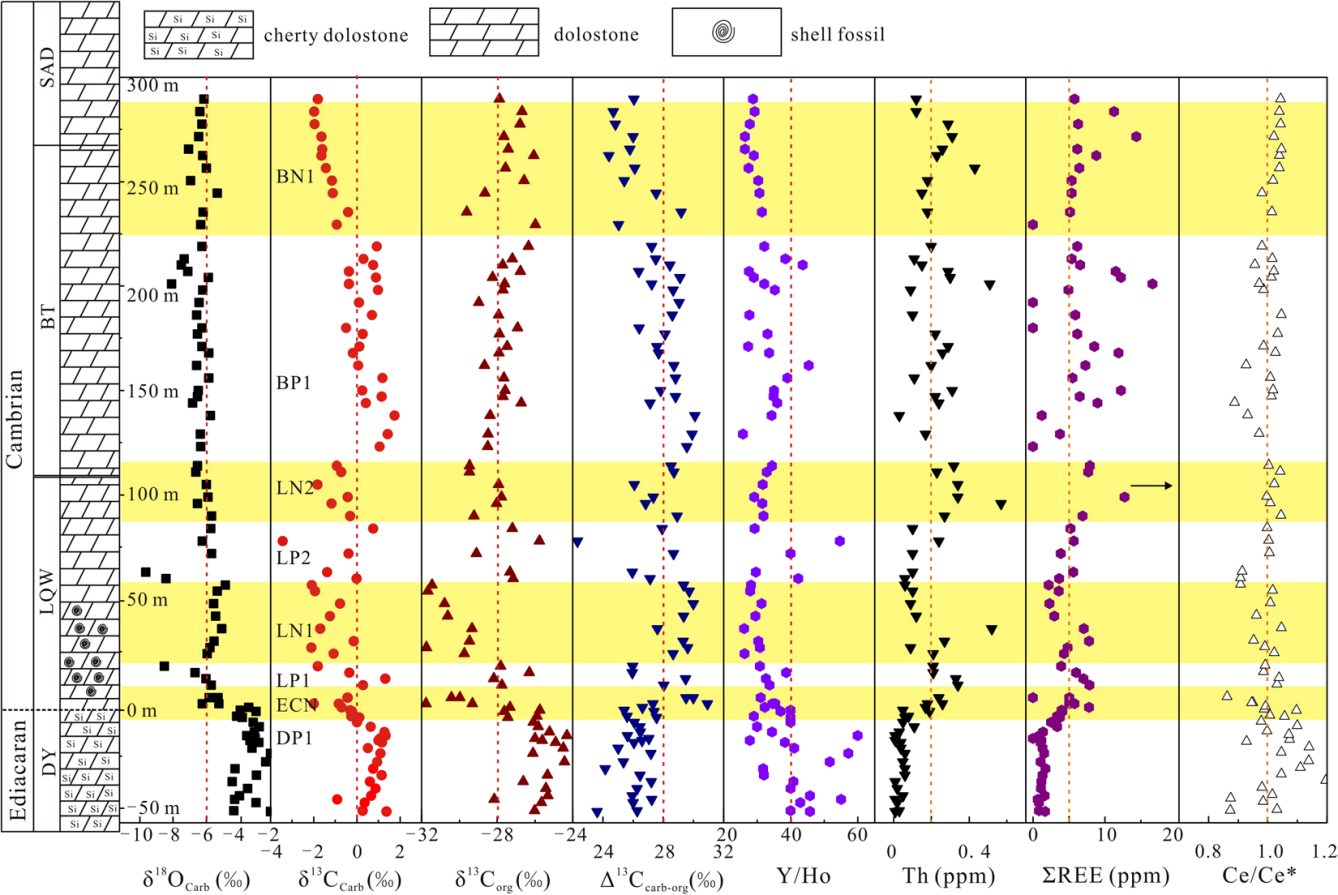
Lithological and geochemical profiles for dolostone from the Ediacaran to early Lower Cambrian strata in the Tangshan section.

The Tangshan section is located in the eastern Anhui of South China (Fig. 1B). The strata there were deposited in a restricted shallow water^39^. From the bottom to the top (Fig. 2), the strata are composed of the Dengying Formation (DY) of the late Ediacaran, and the Lengquanwang Formation (LQW), the Bantang Formation (BT) and the Shanaoding Group (SAD) of the Cambrian^40^. Small shelly fossils, including *Anabarites*, have been found at the base of the LQW Formation, indicating that the E-C boundary is roughly between the DY and LQW formations.

The Lower Cambrian strata in South China are poor dated by isotope geochronology. The highly metalliferous black shales with high Ni-Mo concentrations from the Niutitang and Xiaoyanxi formations was once considered as close to the E-C boundary, but recent radiometric ages of 539.4 ± 2.9 Ma^41^, 532.3 ± 0.7 Ma^42^ and 536.3 ± 5.5 Ma^34^ from ash beds below the highly metalliferous black shales indicate that they are younger than the E-C boundary at 541.0 ± 1.0 Ma. The onset of the globally negative δ^13^C excursion at the lowermost Cambrian strata starts at the top of the Dengying Formation, referred to as the Basal Cambrian C-isotope Excursion (BACE)^43–46^, was constrained at 541.00 ± 0.13 Ma in Oman^47,48^.

## Results

**Figure 3:**
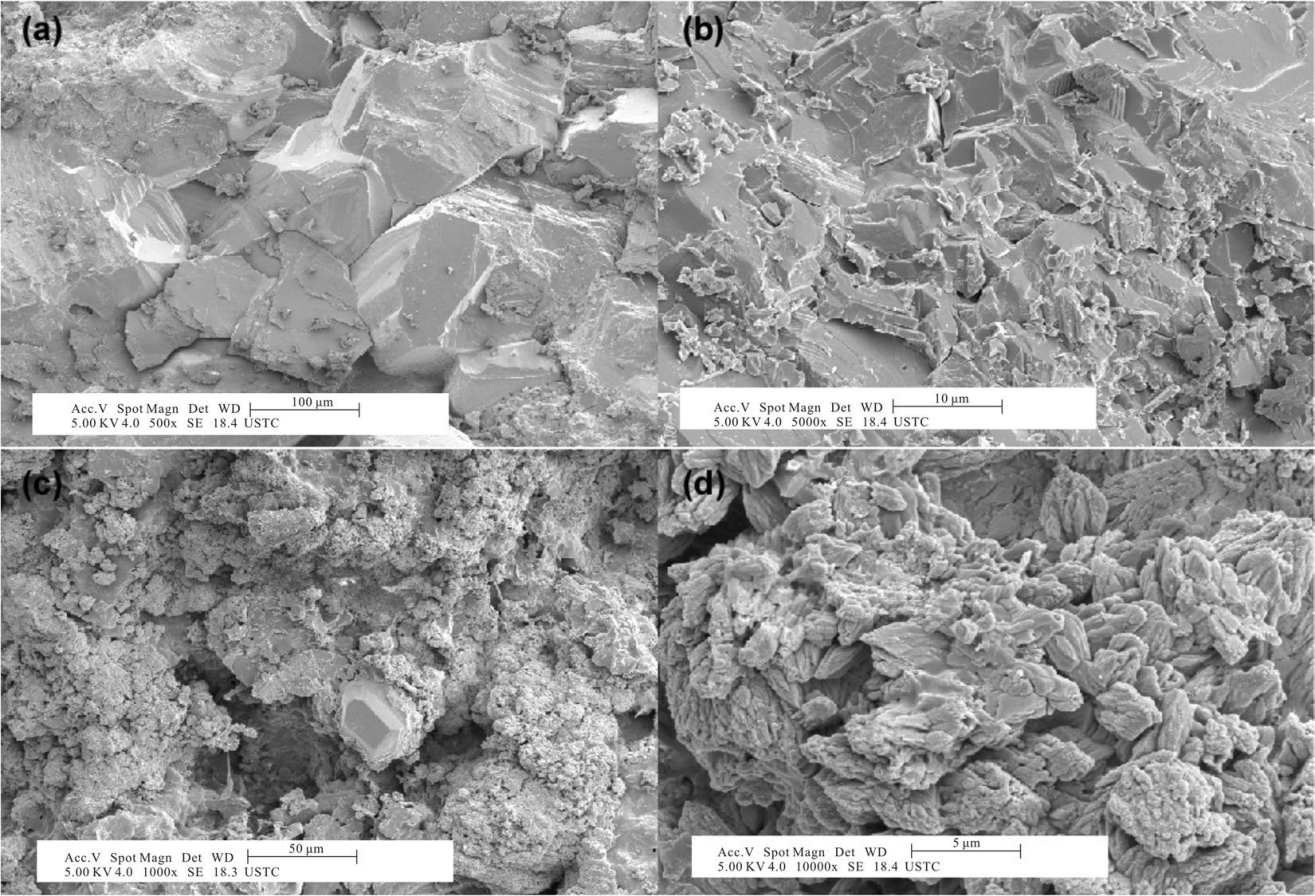
The SEM photograph of 12CH128 in the early Cambrian, (**a**) and (**b**) the compacted micrystalline dolomite. (**b**) and (**c**) the different forms of autogenetic calcite filled in the dolostone.

**Figure 4:**
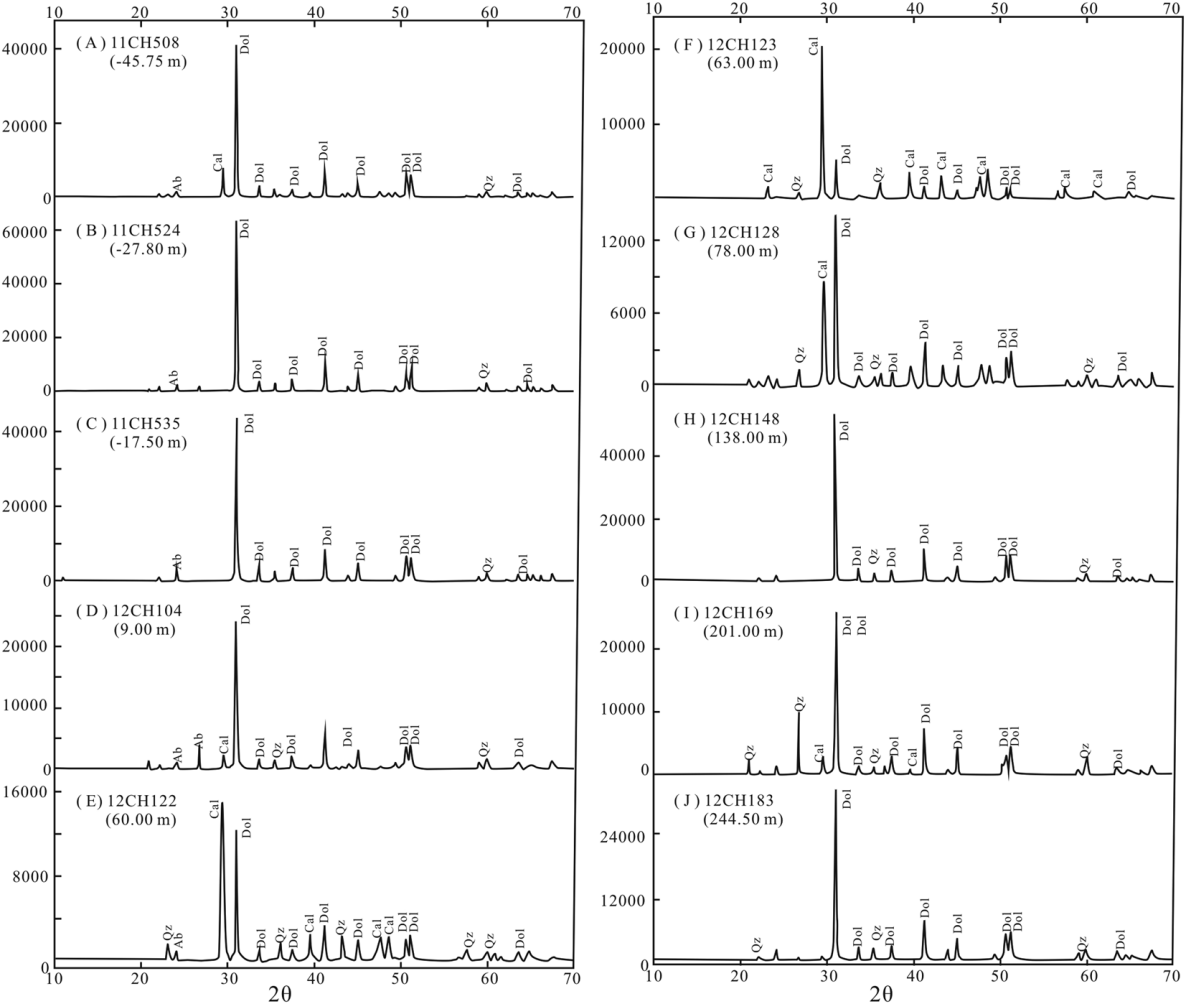
XRD results of the selected dolostone samples on the Tangshan section. The dolomite and calcite have characteristic peak below 30 and above 30, respectively. Dolostones 12CH122, 12CH123 and 12CH128 comprise much calcite crystal in the whole power.

Petrographic observations indicate that most samples on the Tangshan section consist of microcrystalline dolomite with Mg/Ca ratios higher than 0.7^19^. The dolostones show minor to dense packing equigranular and anhedral crystals under a microscope and in SEM images (Fig. 3a,b), indicating primary dolomite textures without significant alteration and metamorphism. Few samples with Mg/Ca ratios lower than 0.7 (Supplementary Table S1) are composed of both calcite and dolomite, as indicated by XRD measurement (Fig. 4). In addition, the SEM image of Sample 12CH128 with a Mg/Ca ratio of 0.38 shows a calcite assemblage among the dolomite grains (Fig. 3c,d), indicating dedolomitization during burial diagenesis.

Variations in δ^13^C_carb_ values and REE + Y patterns for the Tangshan section have been discussed by Li *et al*.^40^ in detail. The δ^13^C_org_ values vary from −31.77 to −22.24‰ (n = 84), with four positive (DP1, LP1, LP2 and BP1) and four negative (ECN, LN1, LN2, and BN1) excursions on the profile (Fig. 2). The δ^13^C_org_ values for the DY Formation in the late Ediacaran vary from −28.15 to −22.24‰ (DP1), and they are positively correlated with the δ^13^C_carb_ values. The first negative δ^13^C_org_ excursion (ECN) is coupled with BACE^43–46^ at the E-C transition (Fig. 3). An overlying positive δ^13^C_org_ excursion (LP1) from −28.17 to −26.29‰ occurs in the lower part of the LQW Formation in the early Cambrian, followed by a negative δ^13^C_org_ excursion (LN1) from −29.32 to −31.66‰. A second positive δ^13^C_org_ excursion (LP2) from −29.22 to −25.75‰ overlies LN1, followed by a third negative δ^13^C_org_ excursion (LN2) from −29.46 to −28.53‰ at the boundary between the LQW and BT formations. Upwards, the δ^13^C_org_ values vary with several δ^13^C_carb_ fluctuations (Fig. 2), around −27.6 to −29.6‰ (including BP1 and BN1 for δ^13^C_carb_) from the upper BT formation to the SAD Formation. The difference between the carbonate and organic carbon isotope compositions (Δ^13^C_carb-org_ = δ^13^C_carb_ − δ^13^C_org_) varies between 22.31‰ and 30.93‰, averaging 22.27‰ (Fig. 2). In particular, the Δ^13^C_carb-org_ values in the early Cambrian LQW Formation (average 28.12‰) are abruptly higher than those in the late Ediacaran DY Formation (average 26.28‰) (Fig. 2).

## Discussion

### Preservation of primary geochemical features

**Figure 5:**
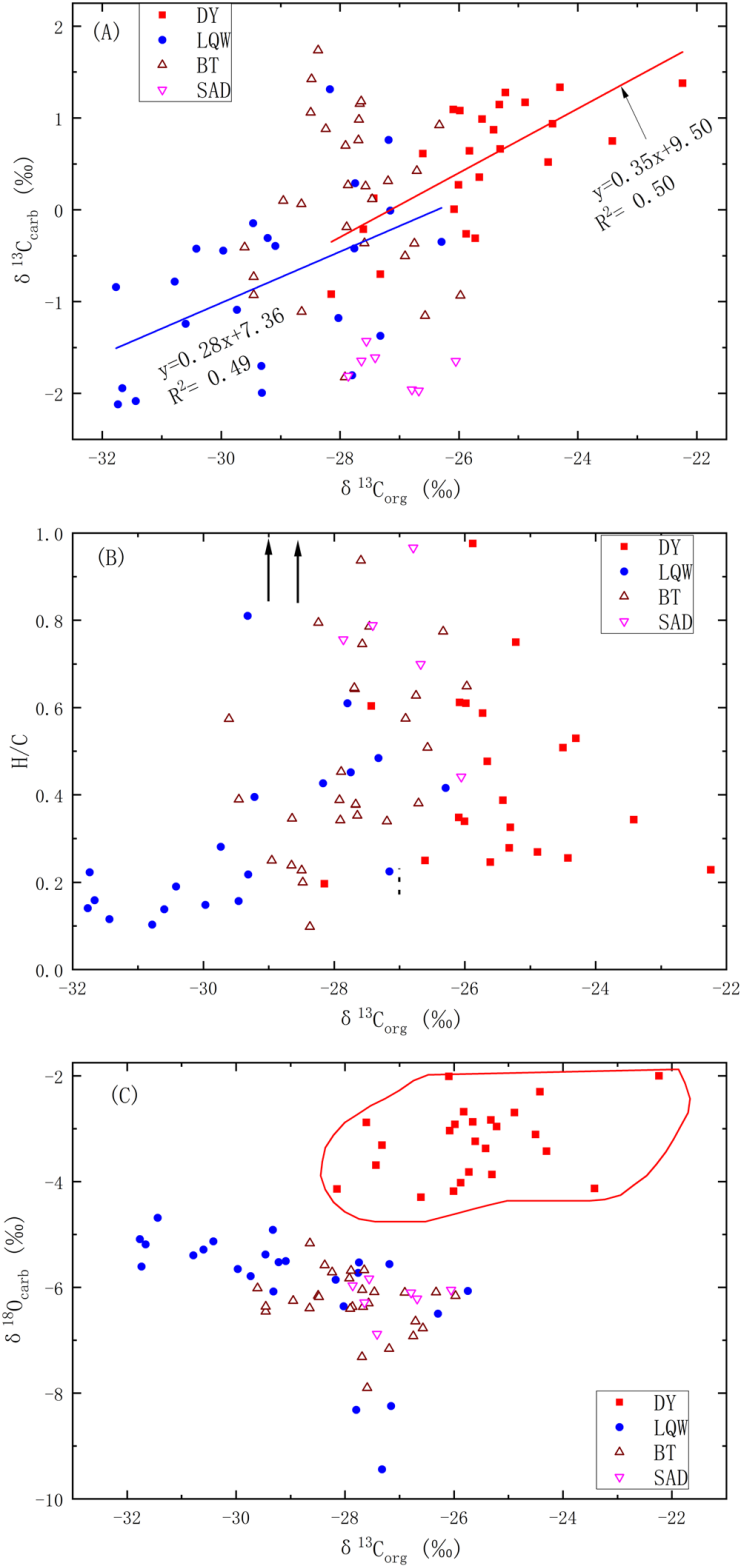
Correlations between δ^13^C_org_ values and (**A**) δ^13^C_carb_ values, (**B**) H/C ratios, (**C**) δ^18^O_carb_ values for dolostone on the Tangshan section. The arrows in (**B**) represent the high H/C ratios for dolostone in the early Cambrian LQW formation.

Most samples from the Tangshan section are composed of dolomite with minor calcite. Dolomite grains show uniform equigranular and anhedral crystals with clear boundary (Fig. 3a,b, and Supplementary Table S1), implying insignificant alteration during post-depositional processes. Several samples were dedolomitized to calcite (Fig. 3c,d). In general, geochemical features such as carbon and oxygen isotope compositions and trace element concentrations are interpreted as primary geochemical signals related to the changes in seawater composition^40^. However, post-depositional processes such as microbial remineralization and diagenetic alteration have a potential to alter the primary δ^13^C_org_ values^49,50^. In particular, the shallow marine carbonates on the Tangshan section could be periodically exposed during sea-level oscillations^40^ and thus influenced by meteoric alteration^50^. Therefore, it is essential to assess the preservation of primary δ^13^C_org_ values. Although the preferential degradation of labile organic compounds can result in an elevation in δ^13^C_org_ values of the residual organic carbon, it is impossible to generate a 4 to 5‰ δ^13^C_org_ change in δ^13^C_org_ values of the residual organic carbon during degradation^51^. In this regard, the δ^13^C_org_ shift from −31.77 to −22.24‰ with a difference as large as 9‰ cannot be produced by the diagenetic alteration alone. In addition, the H/C ratios are higher than 0.2 (except several samples in the LQW and BT formations) and there is no correlations between H/C and δ^13^C_org_ (Fig. 5, and Supplementary Table S1), indicating insignificant effect by the diagenetic alteration.

### Carbon isotope chemostratigraphy

The E-C boundary is defined by the first appearance of small shelly fossils, *Treptichnus Pedum*^52–54^. However, the relationship between the Ediacaran and Cambrian biotas is poorly known due to stratigraphic gaps, taphonomic bias, the restriction of metazoans to oxygenated habits, and the difficulty in integrating commonly disparate bio- and chemostratigraphic data^5,31,46,55^. The BACE, on the other hand, was followed by the rapid appearance and diversification of bilaterian animals in the early Cambrian according to a combined study of lithology, fossils and geochemical data^5,47^. Thus, it can be constrained and better correlated within the interval of characteristic Cambrian-type skeletal fossil distribution^5,34,38,46,56,57^.

The E-C successions on the Tangshan section can be divided into eight δ^13^C_carb_ intervals, from bottom to top named DP1, ECN, LP1, LN1, LP1, LN2, BP1 and BN1^40^. The δ^13^C_org_ values increase from −28.15 to −24.50‰ (DP1) in the Dengying Formation, which can be correlated in trend and magnitude with those of the Ganziping section in western Yunnan^34^, the Jiuqunao, Hezi’ao and Jijiapo sections in Hubei^58^. The DP1 are higher than the contemporaneous values for the Shantan section, the Songtao section^49^, the Longbuzi section^59^, the Yuantuwan-Lijiatuo section^49^ and the Ara Group^60^.

**Figure 6:**
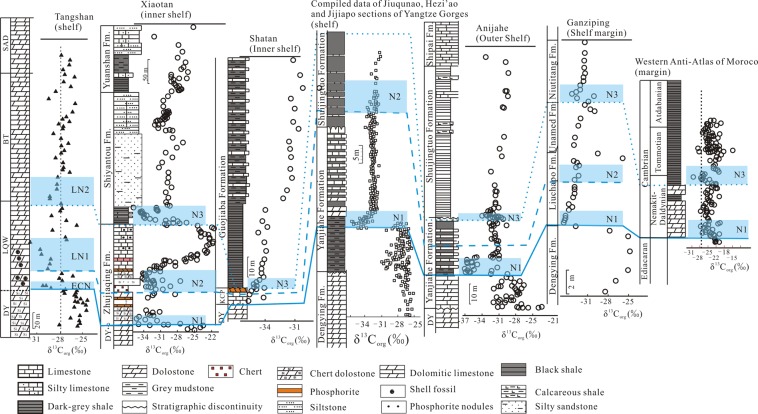
Variations in organic carbon isotope compositions across the Ediacaran-Cambrian transition on the Tangshan section and their stratigraphic correlations with those at Xiaotan^57^, Shatan^49^, Anjiahe^38^, Ganziping^34^, Yangtze Gorge^58^ and the western Anti-Atlas margin of Morocco^46^.

The δ^13^C_org_ excursions are coupled with the δ^13^C_carb_ excursions in the late Ediacaran DY and the early Cambrian LQW formations (Fig. 5A). ECN, a negative δ^13^C_org_ excursion of −4.4‰ from −27.32 to −31.77‰ (Supplementary Table S1), can be identified at the E-C transition (Fig. 2), which can be correlated with the N1 at Xiaotan^57^ and Shatan^49^ of inner shelf, Anjiahe of outer shelf^38^ and Ganziping of shelf margin^34^. Corresponding shifts can also be found in the Jiuqunao, Hezi’ao and Jijiapo sections on the Yangtze platform^56^ (Fig. 6) and a shallow-water section in the western Anti-Atlas margin of Morocco^46^. The worldwide ECN or N1 negative δ^13^C_carb_ excursion, named the BACE^5,45^, is consistent with the first appearance of small shelly fossils of *Anabarites trisulcatus zone*^33^, which was calibrated as the beginning of Cambrian^61^.

Above the ECN (or BACE), a first large positive excursion in both δ^13^C_carb_ and δ^13^C_org_ (LP1) occurred (Fig. 5), followed by a second negative excursion in both δ^13^C_carb_ and δ^13^C_org_ (LN1). The ECN and LN1 can be correlated with the shallow-water carbonates from the other sections on the Yangtze platform (Fig. 6), but they may be lost on some sections due to the unconformity and stratigraphic condensation in shallow-water settings^37,62^. The δ^13^C_carb_ excursion in LN1 can be correlated with Cycle III on the Dovortsy section in Siberia^63^, N2 on the Jiuqunao section in Yichang^58^ and the Anjiahe section in Yangtze Gorge^38^, N2i on the Xiaotan section in Yunnan^64^ and N2 on the Jiuqunao, Hezi’ao and Jijiapo sections^58^. Upwards, the second positive δ^13^C_carb_ and δ^13^C_org_ excursions (LP2) occurred and were overlain by the third negative δ^13^C_carb_ and δ^13^C_org_ excursions (LN2), which can be correlated with N3 on the Anjiahe section^37^, C4^57^ or N3 on the Xiaotan section^37^, N3 on the Shatan^49^, Daotuo^37^, Longbizui^34^ and Yuanwutan sections^49^. In the present study, the three negative δ^13^C_carb_ and δ^13^C_org_ excursions (ECN, LN1 and LN2) are identified in the early Cambrian (Fig. 6). It should be noted that there is also a small negative δ^13^C_carb_ excursion in the SAD Formation, but the δ^13^C_org_ values are relatively stable (Fig. 2).

In addition, the δ^13^C_org_ values largely vary between the different sections because C_org_ can be dominated by local influences associated with the mechanism and rate of carbon fixation, the degree of remineralization, terrestrial organic influx, and trophic structure^38^. Existing δ^13^C_org_ values from the earliest Cambrian in South China show a large variation from −31 to −37‰ (Fig. 6)^34,38,49,57,58^.

### The Δ^13^C_carb-org_ between carbonate and organic matter

**Figure 7:**
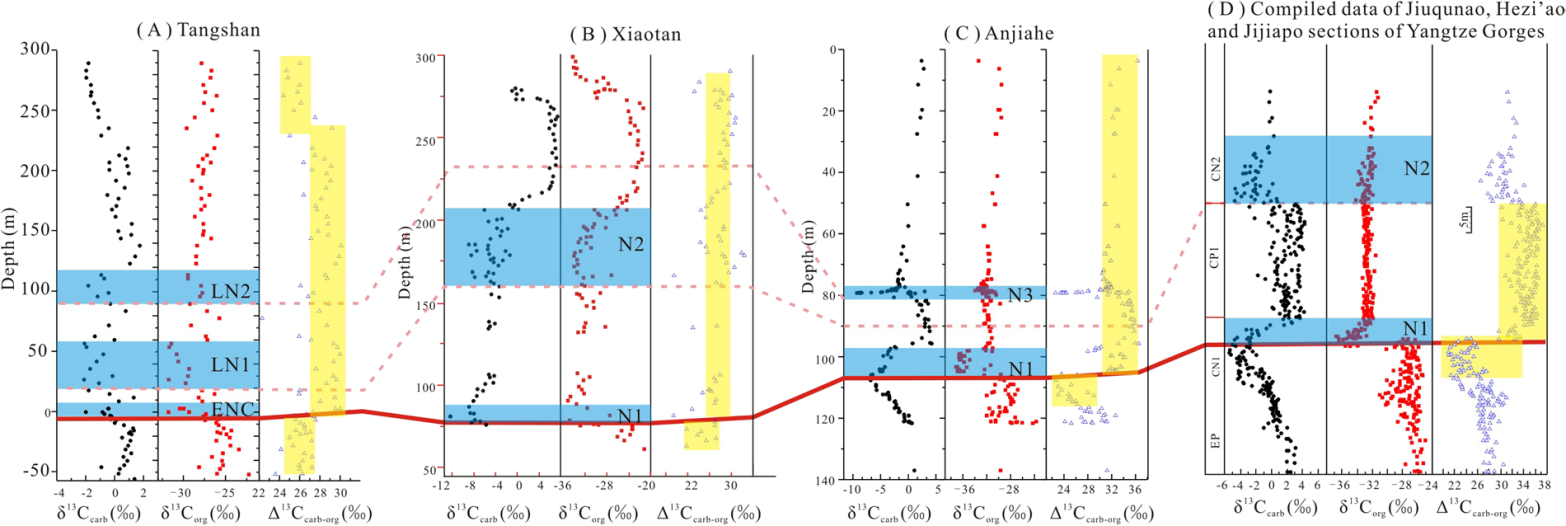
Plot of abrupt change in Δ^13^C_carb-org_ values for the Tangshan section (this study), the Xiaotan section^57,64^, the Anjiahe section^38^ and the compiled data for Jiuqunao, Hezi’ao and Jijiapo sections at the Ediacaran-Cambrian boundary (the solid red line). The yellow areas indicate the variation of Δ^13^C_carb-org_ values, and the blue areas are the correlation between δ^13^C_org_ values in Fig. 6.

The δ^13^C_carb_ and δ^13^C_org_ values are coupled in the late Ediacaran DY Formation and the early Cambrian LQW Formation, but decoupled in the BT and SAD formations (Fig. 5A). In particular, the Δ^13^C_carb-org_ values in the early Cambrian LQW Formation (average 28.12‰) are considerably higher than those in the late Ediacaran DY Formation (average 26.28‰) (Fig. 7), which also occurs on the other contemporary successions although the absolute Δ^13^C_carb-org_ values vary with depositional settings (Fig. 7).

The evidence that the evolution of oxygenic photosynthesis had occurred by ~2.78 Ga is provide by the presence of 2-α methylhapanes from O_2_-producing cyanobacteria^65^ and sterols from O_2_-requiring eukaryotes^66^. Shortly before the Neoproterozoic glaciations, a significant diversification of eukaryotes occurred^67^. In the narrow time interval between the Sturtian and Marinoan glaciations, the steroid diversity and marine planktonic algae had a rapid rise^68^. The rise of algae would create nutrient, energy and photosynthesis, driving ecosystems towards larger and increasing complex organism^68^ and huge environmental changes^15,17,20^. Cultures show that the Δ^13^C_carb-org_ values of marine algae are controlled by the environmental factors, such as temperature, growth rate, pH, CO_2_ and O_2_ concentration^69^. It was concluded that the Δ^13^C_carb-org_ values between CO_2_ and cells are constant (~19‰) from 10 °C to 30 °C^70^. Seawater temperatures were proposed to be lower than 34 °C during the Cambrian^71,72^. In this regard, the temperature effect on Δ^13^C_carb-org_ values between CO_2_ and cells can be negligible during the E-C transition. It is shown that a 2.7‰ change in carbon isotope fractionation can be associated with 0.1 shift in pH values^69^, which is dependent upon P_CO2_ and the influx of alkalinity. However, the [CO_3_]^2−^ concentrations are relatively stable in the early Cambrian^29^. Therefore, the effect of pH variation on Δ^13^C_carb-org_ values may not be important during the E-C transition.

Different photosynthesis species may be a factor related to ^13^C/^12^C fractionation, which may result from differences in growth rates and/or particulate organic carbon production^3^. Several experiments have indicated that the carbon isotope fractionation in algae or plants is primarily driven by the enzyme ribulose-1,5-bisphsphate Carboxylase/Oxygenase (RubisCO)^73^, which is responsible for the fixation of CO_2_ into organic compounds. The carbon fractionation between marine species and the partial pressure of atmospheric carbon dioxide (*p*CO_2_) is clearly CO_2_-dependent, with higher *p*CO_2_ leading to higher carbon isotope fractionation, which have been tested by dinoflagellate species^3^ and coccolithophorid with different growth rates^4^. Intrinsic carbon isotope fractionations associated with RubisCO have been estimated to be ~22‰ to 30‰^74^. It has been shown that the δ^13^C values of *p*CO_2_ are associated with the carbon isotope composition of carbonate^75^. The atmospheric concentration of CO_2_ gas is kept near the Henry’s Law equilibrium with surface seawater [HCO_3_]^−^ through gas exchange during turbulent mixing^75^. Considering the limited variations in the pH, temperature and atmospheric pressure during the E-C transition^29,71,72^, the equilibrium constant of carbon isotope exchange between gaseous and dissolved CO_2_ varies in a small range^76,77^. Thus, the limited carbon isotope fractionation between the atmospheric CO_2_ and the marine dissolved inorganic carbon can be expected. The ^13^C-discrimination between carboxylating reactions (RubisCO) and atmospheric CO_2_ would likely be the reason for the abrupt changes in Δ^13^C_carb-org_ during the E-C transition. However, the enhanced chemical weathering rate from the late Ediacaran DY Formation to the early Cambrian LQW Formation would cause a sudden CO_2_ drawdown and cooling, which is recorded by the CIA index^30,37,78,79^. It has been proven that the decrease of *p*CO_2_ would have induced a decrease in the Δ^13^C_carb-org_ values^3,4^. In this way, the widespread and abrupt increase in Δ^13^C_carb-org_ values during the E-C transition would not result from the variation in *p*CO_2_ (Fig. 7).

The lowered δ^13^C_org_ value can also be induced by the methanogenesis^80^ and the elevated Δ^13^C_carb-org_ excursion could be caused by the methanotrophic biomass^81^. However, the anaerobic carbon monoxide dehydrogenases (CODH) occur under strongly anoxic conditions^82^. In addition, the least oxygenation of surface seawater^5,13–17^ and the model calculations^83^ indicate the sulfate concentrations during the early Cambrian were relatively high, which would reduce the role of methanogenesis. Thus, this possibility can safely be ruled out.

After the other environmental factors have been fully excluded, the best explanation for the abrupt increase in Δ^13^C_carb-org_ values during the E-C transition is a rise in atmospheric O_2_. The dependence of biological carbon isotope fractionation on the partial pressure of atmospheric oxygen (*p*O_2_) has been demonstrated in laboratory experiments with vascular land plants, bryophytes, and marine phytoplankton^84–86^. Increased carbon isotopic discrimination for light isotopes (^12^C) is observed at increasing O_2_ levels because atmospheric CO_2_ levels rise within the plant of plankton cell as photorespiration begin to outpace photosynthesis, which in turn increases the carbon isotope fractionation during growth^85^. In this way, the *p*O_2_ increase can be expected by the increase of Δ^13^C_carb-org_ values though the absolute values of *p*O_2_ are notoriously difficult to track^87^. Beerling *et al*.^85^ have modeled the atmospheric O_2_ dependence of the carbon isotope fractionation between fossil organic matter and CO_2_ (equation 4 in their paper), which was modified by Saltzman, *et al*.^88^ as:

1 $${{\rm{\Delta }}}^{{\rm{13}}}{{\rm{C}}}_{{\rm{meas}}}={{\rm{\Delta }}}^{{\rm{13}}}{{\rm{C}}}_{{\rm{initial}}}+{\rm{J}}\ast [\frac{{{\rm{M}}}_{{\rm{o2}}}}{38}]-{\rm{1}}$$

where Δ^13^C_meas_ is the measured value for the difference between δ^13^C_carb_ and δ^13^C_org_, and Δ^13^C_initial_ is an estimate of the baseline value. In the present study, the average Δ^13^C_carb-org_ value of 26.08‰ for the late Ediacaran DY formation is taken as the Δ^13^C_initial_ value and the average Δ^13^C_carb-org_ values of 28.07‰ for the early Cambrian LQW and BT formations are taken as the Δ^13^C_meas_ value. A value of J = 5 is selected in the calculation based on the best fit to both the experimental data for isotopic discrimination in modern marine phytoplankton and the results of isotope mass balance model^88^. Then, the abrupt increase in the Δ^13^C_carb-org_ values of approximately +2‰ (Fig. 7) indicates that the mass of oxygen in the atmosphere was doubled in the early Cambrian^85,88^. Although oxygen levels during the Late Neoproterozoic are difficult to constrain, it has been proposed that the oxygen levels are higher than 8% to 15% PAL near the end of the Neoproterozoic^89^. In this regard, the *p*O_2_ of early Cambrian would be at least 16 to 30% PAL, which is consistent with 10% to 40% PAL estimated for the Cambrian^87^. The decrease of Δ^13^C_carb-org_ values in the SAD Formation, equivalent to the middle Tommotian (Fig. 6), could be generated by another shift in *p*O_2_ or *p*CO_2_^84,85,90^ or the occurrence of new photosynthetic species^91^.

### Continental trace element influx

**Figure 8:**
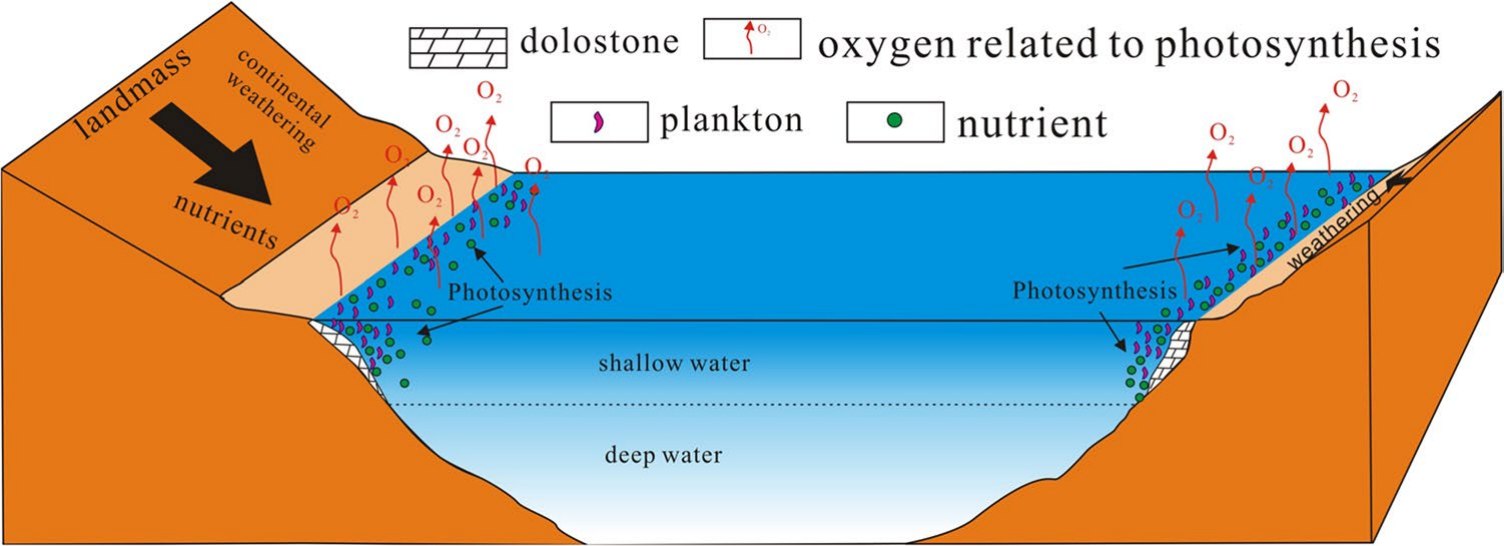
A conceptual model for the emergence of huge landmass and enhanced weathering, which results in the global scattering of nutrients into the seawater through river drainage and wind. This model coincide with the rise of atmospheric *p*O_2_ due to the photosynthesis in the shallow seawater during Ediacaran-Cambrian transition.

During the E-C transition, a number of continental blocks were converged toward a large and complex continent, Gondwana^27,92,93^. In general, this supercontinent is considered as an assembly of four major continental blocks along three collisional orogens^27^. The convergent plate boundaries along the Gondwana-forming sutures could have served as effective sources for nutrient delivery^94^. An unconformity was formed globally^62,95^, followed by a worldwide transgression in the early Cambrian^36^, which would expand the area of shallow epicontinental seas, leading to an increased accommodation space and a sustained flux of continental weathered products to the seawater (Fig. 8). This unconformity is recorded by shallow-water marine carbonates directly overlying on the continental crystalline basement, or basin/slope organic-rich shales/chert unconformably overlying the Ediacaran carbonate shelf at the Shatan and Anjiahe sections, but it is not recorded by those in deep-water sequences on the Longbizui and Yanwutan sections^34,37,49,58,96^. The geochemical features, such as δ^18^O_carb_ values, REE + Y patterns, water-insoluble element concentrations and Y/Ho ratios, suggest that the dolostones of the late Ediacaran DY Formation were deposited from normal seawater^97,98^, but those of the early Cambrian LQW Formation were influenced by a large amount of continental weathered materials, which is consistent with a rapid rise in Sr isotope ratios^99–102^. A number of elements, such as Be, Al, Sc, Co, Ga, Cs, REE, Hf and Th, are water-insoluble during chemical weathering given that they are concentrated in refractory minerals or strongly absorbed by clays^103^. After intensive weathering, these elements were enriched in weathered products, which would be concentrated in the impure freshwater, in river, stream and lake^104^, and ultimately into the seawater. In this way, the element concentrations and patterns of near-shore seawater would be altered by even a minor amount of terrigenous materials because the element compositions of terrigenous materials are quite different from that of seawater^104,105^. The weathered products, including a widespread nutrient supply, such as P, K, Fe, Ca, Mg and others^106^, could be transported to the shallow seawater via aeolian and fluvial systems across the global. Especially, the weathering flux of P into the seawater would increase, which could have promoted the activity of photosynthesis, leading a rise in atmospheric O_2_^28,107^.

### Implications for coincidence in continental weathering and oxygen rise

The early Cambrian witnessed the explosive radiation of animals^5,22,56^. Complex life requires two critical ingredients: nutrient and oxygen. Geological and geochemical studies indicate a constant increase of the oxygen level and a contraction of anoxic seawater during the E-C transition^5,13–18^. It is hypothesized that the oxygenation was triggered through increased weathering fluxes^21,28^, leading to an increase in oceanic primary production and organic carbon burial^28,30,108^. The species engaging in ecosystem engineering might also be responsible for the oxygenation^109,110^.

The emergence of huge landmass and the enhanced weathering of continental crust would transfer global scattering nutrients into the seawater through wind and river drainage in the early Cambrian (Fig. 8), which is indicated by the decrease of Y/Ho ratios and the increase of Th, V, Sc and REE concentrations (Fig. 2 and Supplementary Fig. S1). Although it is difficult to accurately constrain the proportions of fresh water in the surface seawater, the relative proportions of freshwater can be obtained on the basis of the δ^18^O_carb_ values, the water-insoluble element concentrations, Y/Ho ratios (Supplementary Fig. S3). The mixing lines between δ^18^O_carb_ values and Y/Ho ratios represent a result of mixing two end-members between seawater and freshwater, where the freshwater contains the terrigenous material from the chemical weathering into the seawater (Supplementary Fig. S3).

The chemical weathering not only serves as a negative feedback for CO_2_, but also provides the nutrients for feeding of the primary producers. A stable supply of essential nutrients would also exert a significant control on the evolution of myriad life forms^27^. Nutrients, such as P, Ca, Fe, Na^+^, K^+^ and Mo, are predominantly enriched in crustal rocks, such as granitoids and andesite^27^. These elements are critically important to build the ‘hard parts’ (bones, shells, teeth), whereas some of the other nutrients are essential for cell metabolism^106^. Enormous nutrient into the seawater would especially feed the planktons in the sea surface^106^, leading to an accelerated burst of photosynthesis^67,111^, which may be the Earth’s only major source of molecular oxygen, an oxygen source strong enough to sustain a major atmospheric oxygen increase^112^. The atmospheric O_2_ could can reach up to 16 to 30% PAL based on the abrupt increase of Δ^13^C_carb-org_ values during the E-C transition (Fig. 7). Although anoxic seawaters may still have existed widely in the early Cambrian^87,113^, evidence from sedimentary Mo concentrations^5,13,14^ and isotopes^15^, U isotopes^16,17^ and its rare earth element concentrations^18^ indicate strong oxygenation of the deep seawater^15^, consistent with the rise of atmospheric oxygen concentrations^113–115^ and the appearance of filter-feeding sponges^30,31,110^.

Although the timing and magnitude of atmospheric O_2_ accumulation are difficult to be accurately constrained, it is supposed to be regulated by erosion and deposition related to tectonic processes^30,116^. An imbalance between the oxygen production and consumption would result in a gradual rise in the oxygen accumulation^30,109,116^. The high *p*O_2_ is necessary to keep the reaction at the seawater-atmosphere interface in order to produce collagen that paste plural cells together. However, even if the *p*O_2_ is high enough, metazoans cannot be synthesized if the nutrient elements that make up the metazoans were not supplied^106^. In this regard, the simultaneously enhanced weathering input would provide necessary nutrients for biological evolution. Therefore, the increasing amount of landmass, the enhancement of chemical weathering on the continental crust, the supply of a large amount of nutrients into the shelf and high oxygen level in the atmosphere all coincide in time for promoting the birth of large multi-cellular animals.

## Conclusions

The Ediacaran-Cambrian (E-C) transition is a period with substantial biospheric, environmental, and tectonic changes. The δ^13^C_org_ profile on the Tangshan section in South China records three negative excursions during the early Cambrian, which can be compared with those of the other sections in the world. The abrupt increase in Δ^13^C_carb-org_ values indicates a rise of atmospheric oxygen concentrations at the E-C boundary. Although there are several δ^13^C_carb_ and δ^13^C_org_ excursions in the early Cambrian, the atmospheric O_2_ retained high during the deposition of the LQW and BT formations. Geochemical proxies, such as REE + Y patterns, water-insoluble element concentrations and Y/Ho ratios, indicate that an enhanced weathering of the continental crust occurred from the late Ediacaran DY Formation to the early Cambrian LQW Formation during the E-C transition. The enhancement of continental weathering delivers nutrients to the seawater for primary producers, which would accelerate photosynthesis and promote the rise of atmospheric *p*O2. The enhanced weathering along with the rise of atmospheric oxygen, may have boosted the emergence of complex life in the early Cambrian.

## Methods

Some samples and the method of trace element concentrations have been described in Li *et al*.^40^. The methods of this study are as following.

### XRD

The Mg/Ca ratios of all samples were analyzed by Li *et al*.^40^. Only several sample powders with special Mg/Ca ratios were measured in the present study by X-ray diffraction (XRD). The XRD analysis was performed at University of Science and Technology of China (USTC) in Hefei using a TTR-III diffractometer (operating at 40 kV, 200 mA) equipped with a fixed graphite monochromator and a Cu target tube in the 2θ with a steep size of 0.02 degree. Meanwhile, the microstructure of the carbonate was observed by the scanning electron microscope (SEM) at USTC.

### Oxygen and carbon isotopes

The methods for the analyses of carbonate C and O isotopes (δ^13^C_carb_ and δ^18^C_carb_) were presented in Li *et al*.^40^. The organic carbon isotope compositions (δ^13^C_org_) were measured on 84 samples. The 50% (v/v) guarantee reagent hydrochloric acid had been used at 60 °C for 24 h to remove the all carbonate components in whole-rock powders, and the undissolved part was washed by ultra-pure water and extracted for three times to achieve neutral solution conditions^19^. The undissolved part of the carbonate were dried at 70 °C and weighed, and then they were analyzed to get the TC, TH and TN of the residue by the Elementar vario EL cube at USTC. The analytical precisions of TC, TH and TN are better than ±0.3%. The organic carbon isotopes of the residues were analyzed via online EA-1112 link to Finigan Delta-Plus XL at State Key Laboratory of Organic Geochemistry in Guangzhou Institute of Geochemistry, Chinese Academy of Sciences (CAS), Guangzhou. The results are calibrated from the standard carbon black δ^13^C_PDB_ = −36.91‰ and given in the standard delta notation as per mil difference to the Vienna PDB standard (VPDB). Reproducibility was generally better than ±0.5‰.

## Supplementary information

Supplementary Figures

Table S1 to Table S7
