# Supplementary material for: Enhanced weathering as a trigger for the rise of atmospheric O2 level from the late Ediacaran to the early Cambrian
Source: Sci Rep. 2019 Jul 23;9:10630. doi: 10.1038/s41598-019-47142-3 (PMC6650434; doi:10.1038/s41598-019-47142-3)
Supplement: Supplementary file 1 — Supplementary Figures [file 41598_2019_47142_MOESM1_ESM.pdf]

Enhanced weathering as a trigger for the rise of atmospheric O_2 level from Late Ediacaran to Early Cambrian

Wei-Ping Li[a], Yan-Yan Zhao[b,c,] *, Ming-Yu Zhao[d], Xiang-Ping Zha[a], Yong-Fei Zheng[a,]*

[a] CAS Key Laboratory of Crust-Mantle Materials and Environments, School of Earth and Space Sciences, University of Science and Technology of China, Hefei 230026, China

[b] Key Lab of Submarine Geosciences and Prospecting Techniques, MOE, Institute for Advanced Ocean Study, College of Marine Geosciences, Ocean University of China, Qingdao 266100, China

[c] Laboratory for Marine Mineral Resources, Qingdao National Laboratory for Marine Science and Technology, Qingdao 266237, China

[d] Department of Geology and Geophysics, Yale University, New Haven, Connecticut 06511, USA

*Corresponding authors. Emails: yanyanzhao@ouc.edu.cn (YYZ); yfzheng@ustc.edu.cn (YFZ)

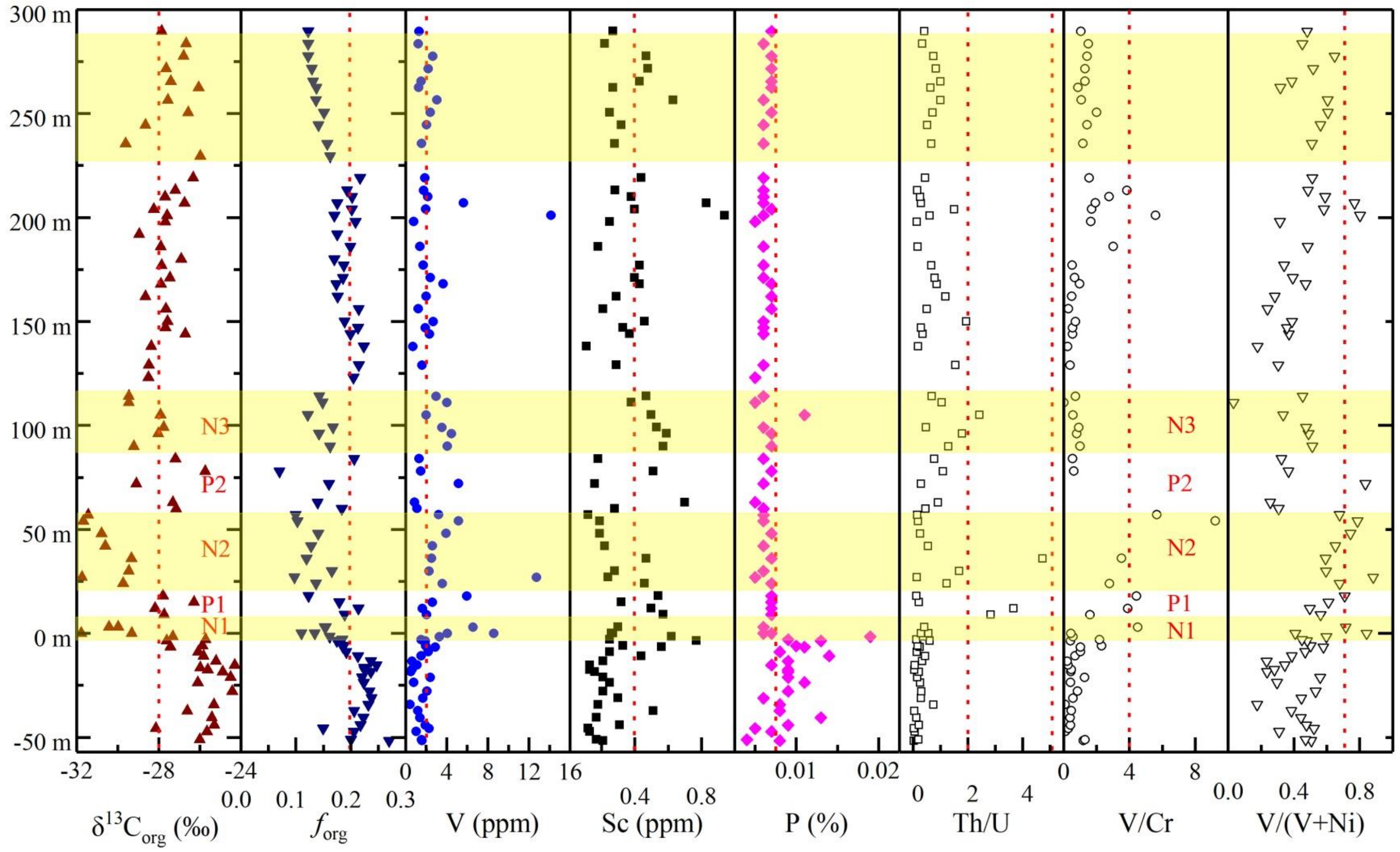

Fig. S1. The geochemical profiles for the dolostone from the Ediacaran to early Lower Cambrian strata on the Tangshan section. f_{org} is calculated based on $\delta^{13}C_{carb}$ and $\delta^{13}C_{org}$.

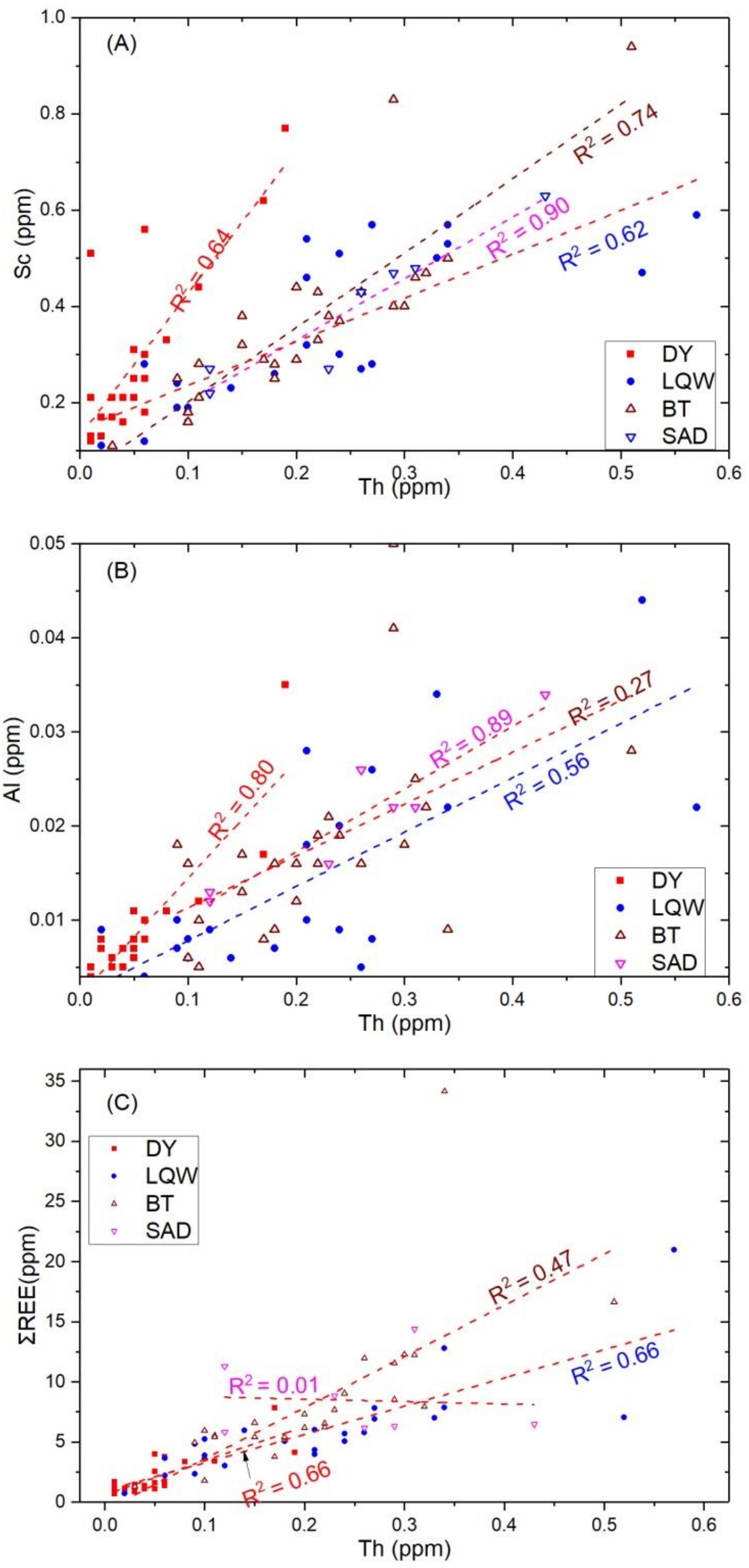

Fig. S2. Correlations between Th concentrations and (A) Sc, (B) Al, (C) REE concentrations of dolostone on the Tangshan section. The positive correlations are common between Th and REE concentrations (except that in the SAD Formation). The profiles of Th and REE concentrations are shown in Fig. 4, representing the variations in the concentration of insoluble elements.

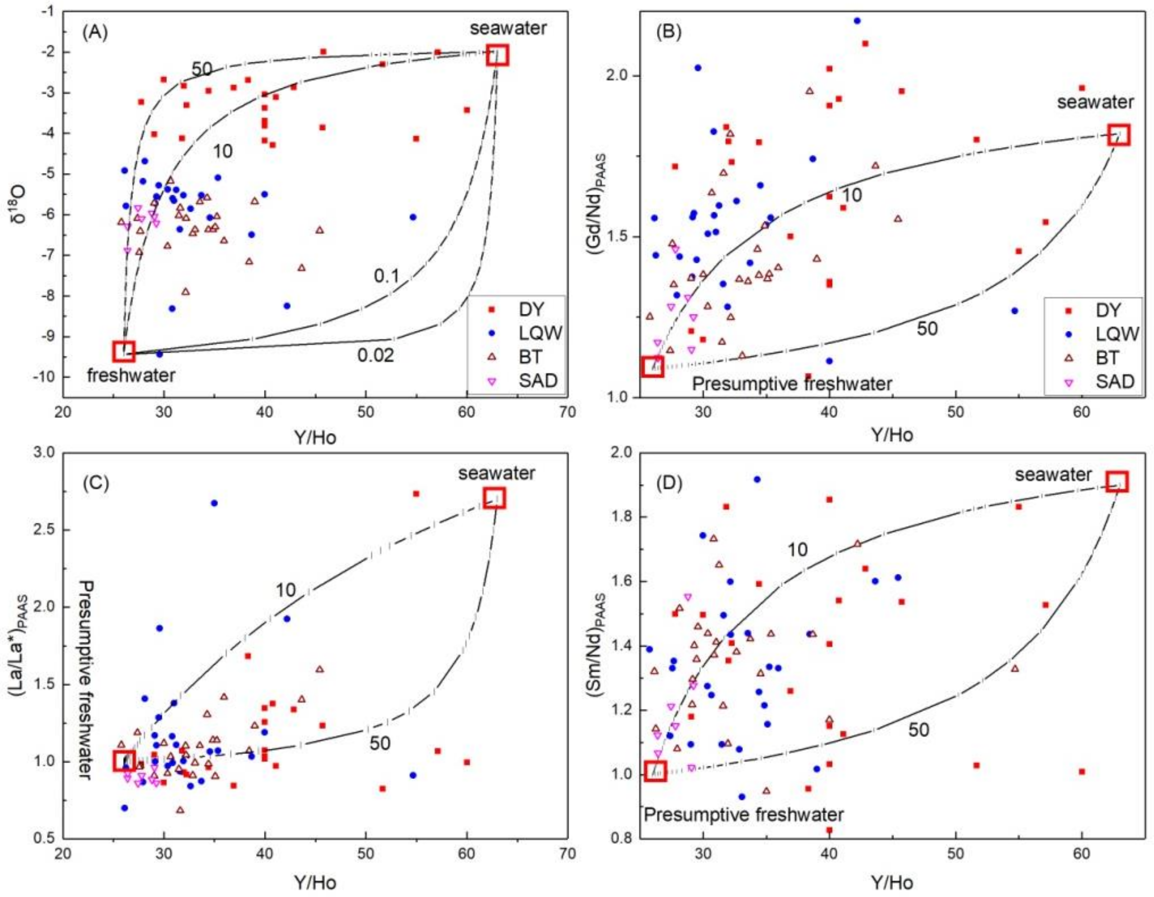

Fig. S3. Cross-plots of Y/Ho and (A) $\delta^{18}O$, (B) $(La/La^*)_{PAAS}$, (C) $(Gd/Nd)_{PAAS}$, (D) $(Sm/Nd)_{PAAS}$ for dolostone on the Tangshan section. Because the REE concentrations of freshwater are generally between 10 and 50 times higher than those of seawater[116], the mixing line represent the mixing of Ho between freshwater and shallow seawater with the ratios of 10 and 50. The carbonate-water partition coefficient of REE + Y in the model is 259.6[117]. The marine carbonate end-member is assumed to have the same Y/Ho and $\delta^{18}O_{carb}$ values as modern marine carbonate, 63 and –2.79‰, respectively, whereas those of the freshwater end-member are assumed to be 26 and –10.46‰, respectively.
